# Corneal keloid: four case reports of clinicopathological features and surgical outcome

**DOI:** 10.1186/s12886-016-0372-4

**Published:** 2016-11-09

**Authors:** Hyo Kyung Lee, Hyuk Jin Choi, Mee Kum Kim, Won Ryang Wee, Joo Youn Oh

**Affiliations:** 1 Department of Ophthalmology, Seoul National University Hospital, 101 Daehak-ro, Jongno-gu, Seoul, 110-744 Korea; 2 Laboratory of Ocular Regenerative Medicine and Immunology, Biomedical Research Institute, Seoul National University Hospital, 101 Daehak-ro, Jongno-gu, Seoul, 110-744 Korea

**Keywords:** Case report, Corneal keloid, Superficial keratectomy, Amniotic membrane transplantation, Mitomycin C

## Abstract

**Background:**

Surgical outcome of corneal keloid is largely variable depending on reports, although surgical management is inevitable in visually significant cases. We here report clinical features, histopathological findings, and surgical outcome of four cases of corneal keloid.

**Case presentation:**

Four Korean male patients without a history of corneal trauma or disease were clinically and histologically evaluated for a slowly-growing, white opacity in the cornea. On slit lamp examination, corneal lesions appeared as a solitary, pearly white, well-circumscribed nodule with a smooth and glistening surface. Because the lesions involved the visual axis deteriorating the visual acuity, the nodules were surgically removed by superficial keratectomy in all patients. Amniotic membrane transplantation was combined in three patients, and an intraoperative mitomycin C application in two patients. Hematoxylin-eosin staining of the excised nodules revealed epithelial hyperplasia, Bowman’s layer disruption, thick and irregularly-arranged collagen fibers in the stroma, and accumulation of prominent fibroblasts, which are consistent with the diagnosis of corneal keloid. The corneal keloids recurred in all patients within 10 months of surgical excision and outgrew the boundary of the excised area.

**Conclusion:**

A diagnosis of corneal keloid should be suspected in patients presenting with an enlarging, white, glistening corneal nodule, even in the absence of a history of corneal trauma or disease. The recurrence is common after surgical excision, and the lesion can be exacerbated by surgery.

## Background

Corneal keloid is a benign proliferation of fibrous or fibrovascular tissue in the corneal stroma, and presents as a single, enlarging, white, elevated, well-circumscribed corneal lesion with glistening surface [1]. Most of corneal keloids develop following a trauma or disease in the cornea, suggesting an aberrant corneal reparative process as one of the factors underlying the pathogenesis of the keloid [1]. However, several cases of primary corneal keloid have been reported in patients without a history of corneal surgery or trauma [2–4]. Surgical management is inevitable in visually significant cases of corneal keloid, but its outcome is largely variable depending on reports.

We herein report the clinical features, histopathology, and surgical outcome of primary corneal keloids in four Korean patients who did not have a history of corneal trauma or disease.

## Case presentation

The study was approved by the Institutional Review Board of Seoul National University Hospital. The patient characteristics were summarized in Table 1.

**Table 1:** Summary of patient characteristics

	Case 1	Case 2	Case 3	Case 4
Sex	Male	Male	Male	Male
Laterality	Right	Left	Both	Right
Age at presentation	21 years	4 years	16 years	62 years
Systemic comorbidity	Osteogenesis imperfecta	Neuro-fibromatosis type 1	-	-
Ocular surgery	Strabismus surgery (both)	Epiblepharon repair (both)	Epiblepharon repair & frontalis sling operation (both)	PRK* (both) Vitrectomy (right)
Age at the time of ocular surgery	5 years	4 years	4 years	44 years (PRK*) 53 years (vitrectomy)

* PRK: Photo-refractive keratectomy

### Case 1

A 21-year-old man presented with a slowly-growing, white opacity in the right cornea. The patient had been diagnosed with osteogenesis imperfecta, and received strabismus surgery in both eyes at the age of five. Otherwise, he denied having a history of ocular trauma, infection or inflammatory disease.

**Fig. 1:**
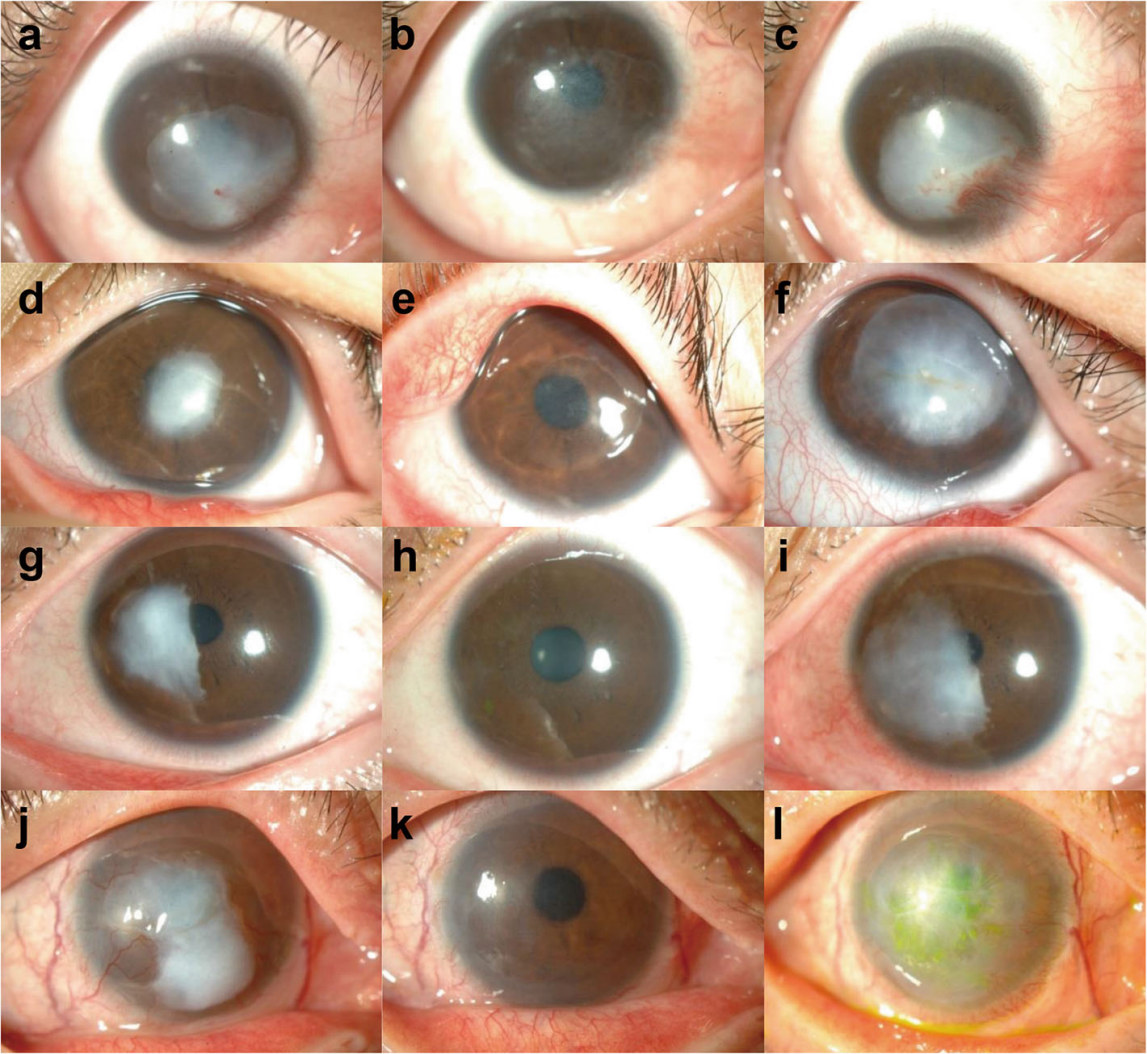
Slit-lamp microscopic photographs of corneal keloids in four patients. **a**-**c** Case 1, (**d**-**f**) case 2, (**g**-**i**) case 3, (**j**-**l**) case 4. Shown are corneal photographs at presentation (**a**, **d**, **g**, **j**), right after superficial keratectomy (**b**, **e**, **h**, **k**), and after recurrence (**c**, **f**, **i**, **l**)

**Fig. 2:**
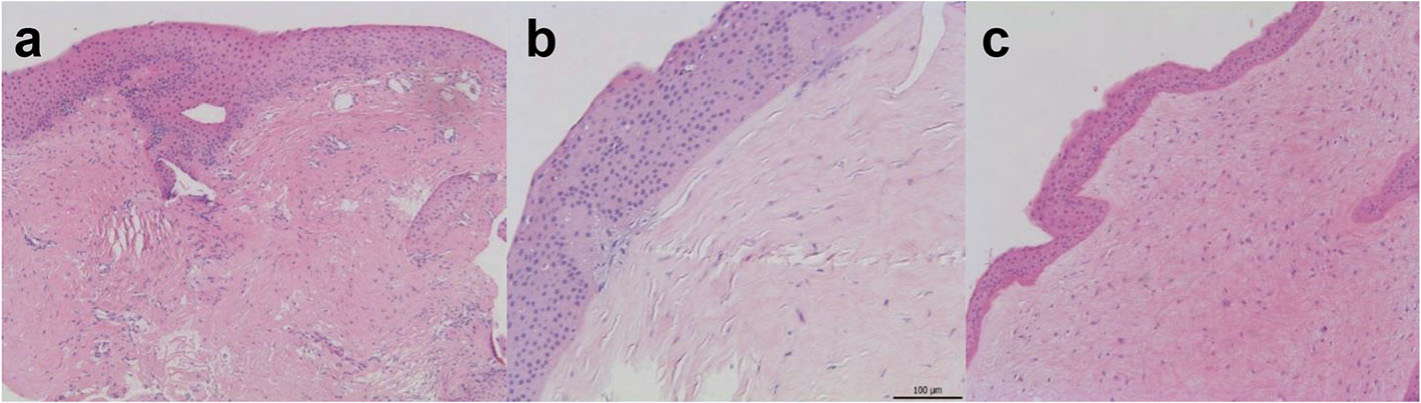
Hematoxylin-eosin staining of the excised keloid sections. **a** Case 1. The epithelium is hyperplastic and undulating, and Bowman’s layer is totally disrupted. The stromal collagen bundles are increased and irregularly-arranged in a whorl-like pattern. Original magnification × 100. **b** Case 2. A marked epithelial hyperplasia and focal disruption of Bowman’s layer are noted. Original magnification × 200. **c** Case 4. Thickened epithelial layer, increased stromal collagen, and numerous accumulation of prominent fibroblasts are observed. Original magnification × 100

On initial examination, the best corrected visual acuities (BCVA) were 20/125 in the right eye and 20/20 in the left eye. Slit-lamp biomicroscopy showed a pearly white, elevated opacity in the right cornea with distinct margins and smooth surface (Fig. 1a). New vessels were partially present in the lesion adjacent to the limbus. The left cornea was clear. The lesion was removed by superficial keratectomy (SK), and the residual stromal bed was temporarily covered with an amniotic membrane (AM). Histopathologic examination of the excised lesion revealed hyperplastic epithelium, the absence of Bowman’s layer, and abundant, erratically-interlaced collagen fibers with prominent fibroblasts in the stroma (Fig. 2a). The clinical and histologic findings were compatible with a diagnosis of corneal keloid [1]. One month after surgery, the patient’s vision improved to 20/50, and the cornea appeared clear except for a fine reticular subepithelial haze (Fig. 1b). However, 4 months later, corneal opacification recurred and worsened along with more severe new vessel ingrowth at the same location as before the surgery (Fig. 1c).

### Case 2

A 4-year-old boy presented with an enlarging white nodule in the left cornea for the past two weeks. He had been diagnosed with neurofibromatosis type 1 at birth, and had a history of craniofacial reconstruction at the age of two. Two months before presentation, the patient underwent lower lid epiblepharon repair in both eyes. There was no other history of ocular surgery, trauma or disease.

Examination revealed that BCVA were 20/40 in the right eye and 20/100 in the left eye. A single, 3-mm-sized, white, avascular nodule was noted at the center of the left cornea, while the right cornea was clear. The nodule was completely excised by SK, and histologic examination of the biopsy specimen showed thick deposition of hyalinized collagen fibers and inflammatory cell infiltrates in the corneal stroma. Four months later, more dense and elevated nodule with a pearly, glistening surface recurred at the site of the previous lesion, outgrowing the initial boundary (Fig. 1d). The vision in the left eye decreased to 20/200. The recurred lesion was successfully removed by a repeat SK with faint stromal haze left (Fig. 1e), and the vision improved to 20/125. Histology revealed a marked epithelial hyperplasia, partially-disrupted Bowman’s layer, and increased collagen deposition in a whorl-like pattern, which are consistent with the diagnosis of corneal keloid (Fig. 2b). However, five months after the repeat SK, the lesion recurred in a more aggravated manner, reducing the vision to hand motion. The third SK was performed to excise the lesion and combined with an intraoperative application of 0.02 % mitomycin C (MMC) for 1 min and an overlay AM transplantation (AMT). Four month later after the third SK, corneal opacification rapidly recurred in a discoid shape which corresponded to the SK site (Fig. 1f).

### Case 3

A 16-year-old man visited the clinic because of white corneal opacities in both eyes. The lesion had been present for 2 years and had progressively enlarged. The patient had lower lid epiblepharon repair and frontalis sling operation in both eyes at the age of four. He had no preceding history of trauma, surgery or inflammatory disorders in the cornea.

Slit-lamp biomicroscopy revealed that a solitary, white, elevated opacity was present in both corneas. The lesion of the left eye was larger than that of the right eye and involved the visual axis (Fig. 1g). Therefore, SK was performed to excise the lesion, which was separated easily from the underlying stroma. After the removal of the mass, 0.02 % MMC was applied to the corneal surface for 30 s, and AM was overlaid to the stromal bed in order to facilitate wound healing and reduce the haze (Fig. 1h). Seven months later, the nodule recurred to the same dimensions as before the SK was performed. The recurred nodule was removed by a repeat SK, and was found to firmly adhere to the underlying stroma during operation. Five months after the second SK, the lesion relapsed again and extended beyond the area of the initial lesion (Fig. 1i).

### Case 4

A 62-year-old man was referred to our clinic for a white nodule in the right eye. The patient had received photorefractive keratectomy in both eyes for high myopia 18 years before, and had vitrectomy in the right eye for retinal detachment associated with macular hole 9 years before. He had no light perception vision in the right eye ever since. There was no other history of corneal trauma or infection.

On examination, a large white nodular opacity was noted in the central cornea, and surrounded by new vessels growing from the limbus and conjunctiva (Fig. 1j). The nodule was removed by SK. Histologic examination of the nodule showed thickened epithelium and irregular cellular arrangement in the basal layer (Fig. 2c). The stroma was edematous, and a number of prominent fibroblasts were present. After the surgery, the cornea appeared clear, and new vessels regressed (Fig. 1k). However, 10 months later, diffuse corneal opacity recurred with ingrowth of limbal new vessels (Fig. 1l).

## Conclusions

Corneal keloid is generally considered to be excessive collagen deposition as a result of deviation in the normal corneal reparative process [1]. Characteristically, corneal keloid extends beyond the area of the initial trauma over time and can develop months and years after the initial insult, as opposed to hypertrophic scar that retracts gradually and appears immediately after a trauma [1]. Most of corneal keloids are secondary to corneal injury, surgery or disease, but there are several reports of congenital and primary corneal keloids without any antecedent insults to the cornea [2–4]. Corneal keloid is usually seen in the first two decades of life, and occurs more frequently in males than in females [1]. We herein reported four cases of corneal keloids in male patients without obvious history of corneal injury or disease. Two of our patients had a history of lid surgery, two months and 12 years before presentation, respectively. Therefore, it is possible that exposure keratopathy might have preceded the development of corneal keloids in these patients.

The diagnosis of corneal keloid is made by clinical and pathological examinations. Clinically, corneal keloid appears as a solitary, pearly white, elevated, localized lesion with a smooth and shiny surface which is well-demarcated from the adjacent cornea [1–4]. New vessels may or may not accompany the lesion. Pathologically, corneal keloid is characterized by hyperplastic epithelium, disorganized basal layer, disrupted Bowman’s layer, and abundant, irregularly-arranged collagen bundles along with activated fibroblasts in the stroma [1–4]. These clinical and pathological findings were observed in our patients, which made the diagnosis of corneal keloid, and were differentiated from Salzmann’s nodular degeneration (SND) because corneal nodules in SND are multiple and smaller (1–2 mm in size) [5]. Also, SND mostly occurs in patients aged 50–60 years, and has a female predominance [5].

Various modalities have been used to treat visually significant corneal keloids, including SK, lamellar (LK) or penetrating keratoplasty (PK), phototherapeutic keratectomy (PTK), sclerokeratoplasty, or keratoprosthesis implantation [1, 2]. Recurrence has been reported after SK, LK, PK, and PTK [2, 6]. To prevent the recurrence, AMT has been combined with SK in several reports [7, 8] because AM has anti-inflammatory and anti-fibroblastic properties. These previous reports suggested AMT was successful in preventing recurrence [7, 8]. However, corneal keloids recurred in all three of our patients treated with SK and AMT. Additionally, we used an intraoperative application of MMC in two patients along with SK and AMT to suppress activated fibroblasts and keratocytes in the residual stroma, but the lesions recurred nonetheless. There are several speculations on the role of vascularization, innervation, or myofibroblastic transformation in the formation and recurrence of a corneal keloid based on histological and ultrastructural studies [8–10]. While diffuse and anterior vascularization of corneal keloids was described in some reports, other reports demonstrated the presence of few vessels in the lesion. In our study, new vessels growing anteriorly from the limbus accompanied corneal keloids both at the time of presentation and recurrence in two out of four patients. Therefore, it is possible that new vessel growth or partial limbal stem cell deficiency might be involved in the pathogenesis of corneal keloid development or recurrence in some cases. Another possibility is that the presence of aberrant nerves and myofibroblasts in the corneal stroma is the cause of nodule recurrence and stromal opacification after keloid excision. In support for this possibility, some authors recently reported patients who were successfully managed with deep anterior lamellar keratoplasty to remove the entire stroma baring Descemet’s membrane [2, 11]. Accumulation of clinical reports and development of experimental models of corneal keloid formation would be essential for a better understanding of pathogenesis underlying corneal keloids.

In summary, corneal keloid should be suspected in patients who present with a single, white, glistening, elevated corneal nodule even in the absence of previous corneal trauma, surgery or disease. The recurrence is very common after surgical removal, and occurs in a more exacerbated manner. Hence, a careful consideration is necessary when deciding on surgical intervention and procedure for corneal keloid.

## Abbreviations

AM: Amniotic membrane
AMT: Amniotic membrane transplantation
BCVA: Best corrected visual acuities
LK: Lamellar keratoplasty
MMC: Mitomycin C
PK: Penetrating keratoplasty
PTK: Phototherapeutic keratectomy
SK: Superficial keratectomy
SND: Salzmann’s nodular degeneration
